# Neuronal chemokine concentration gradients mediate effects of embryonic ethanol exposure on ectopic hypocretin/orexin neurons and behavior in zebrafish

**DOI:** 10.1038/s41598-023-28369-7

**Published:** 2023-01-26

**Authors:** Adam D. Collier, Nushrat Yasmin, Olga Karatayev, Abdul R. Abdulai, Boyi Yu, Nailya Khalizova, Milisia Fam, Sarah F. Leibowitz

**Affiliations:** Laboratory of Behavioral Neurobiology, The Rockefeller University, 1230 York Avenue, New York, NY 10065 USA

**Keywords:** Neuroscience, Neurodevelopmental disorders

## Abstract

Embryonic ethanol exposure in zebrafish and rats, while stimulating hypothalamic hypocretin/orexin (Hcrt) neurons along with alcohol consumption and related behaviors, increases the chemokine receptor Cxcr4 that promotes neuronal migration and may mediate ethanol’s effects on neuronal development. Here we performed a more detailed anatomical analysis in zebrafish of ethanol’s effects on the Cxcl12a/Cxcr4b system throughout the entire brain as it relates to Hcrt neurons developing within the anterior hypothalamus (AH) where they are normally located. We found that ethanol increased these Hcrt neurons only in the anterior part of the AH and induced ectopic Hcrt neurons further anterior in the preoptic area, and these effects along with ethanol-induced behaviors were completely blocked by a Cxcr4 antagonist. Analysis of *cxcl12a* transcripts and internalized Cxcr4b receptors throughout the brain showed they both exhibited natural posterior-to-anterior concentration gradients, with levels lowest in the posterior AH and highest in the anterior telencephalon. While stimulating their density in all areas and maintaining these gradients, ethanol increased chemokine expression only in the more anterior and ectopic Hcrt neurons, effects blocked by the Cxcr4 antagonist. These findings demonstrate how increased chemokine expression acting along natural gradients mediates ethanol-induced anterior migration of ectopic Hcrt neurons and behavioral disturbances.

## Introduction

Exposure to alcohol during early development produces long-lasting neuronal and behavioral disturbances in the offspring. Recent studies in rodents show that embryonic exposure to ethanol at low-moderate doses increases alcohol consumption later in development. This behavioral change is found to correlate with an increase in number of hypocretin/orexin (Hcrt) expressing neurons in the lateral hypothalamus, a neuropeptide known to promote alcohol intake and related behaviors including arousal, anxiety and motivation^1,2^. These phenomena are also evident in zebrafish, a species with high physiological and genetic homology to humans, a comparable CNS that develops rapidly^3^, and a sophisticated behavioral repertoire^4^. Embryonic exposure of zebrafish to ethanol in the water at low-moderate doses has behavioral effects similar to those produced by prenatal ethanol exposure in rodents and humans, including an increase in alcohol intake^5–7^, and these ethanol-induced behaviors are accompanied by an increase in number of Hcrt neurons in the anterior hypothalamus (AH) where they are normally located. By altering the timing and directionality of cellular migration^8,9^, ethanol exposure can cause neurons to end up in ectopic brain areas outside of their normal location^10^, resulting in the formation of heterotopias^11^, as reported in the brains of children with fetal alcohol spectrum disorder (FASD)^12^.

There is evidence that the neuroimmune system has an important role in controlling neuronal development under normal conditions. Studies of the chemokine ligand Cxcl12 and its primary receptor Cxcr4 demonstrate their function in mediating neurogenesis, proliferation, and migration of neurons^13,14^, with their expression in both rodents^15,16^ and zebrafish^17,18^ often occurring naturally along concentration gradients where higher levels of Cxcl12 attract cells that express Cxcr4. In mice, Cxcr4 deficiency leads to a reduction in number and impaired migration of gonadotropin-releasing hormone (GnRH) neurons^19,20^ and also causes an inappropriate accumulation of dopaminergic neurons^21^. In zebrafish, Cxcr4-mediated signaling along concentration gradients guides the migration of various types of cells, including posterior lateral line primordia^22^, primordial germ cells^23^, and trigeminal sensory neurons^24^. Perturbation of Cxcl12 expression gradients disturbs neuronal development, with Cxcl12 null mice exhibiting a decrease in number of GnRH neurons and inhibition of their migration^20^ and knockdown of Cxcl12 in zebrafish interrupting the migration of GnRH neurons^25^. These studies demonstrate how chemokines are critical for normal neuronal development, with disturbances markedly altering migration.

Recent studies in this lab have provided some evidence in both rats and zebrafish suggesting the involvement of chemokines in the stimulatory effects of ethanol on neuronal development. They show that embryonic ethanol exposure at low-moderate doses increases Cxcr4 receptors in Hcrt neurons and hypothalamic neuroprogenitor cells in rats^26^. Also, ethanol at low-moderate doses increases *cxcl12* and *cxcr4* transcripts in the hypothalamus and internalization of Cxcr4 in zebrafish, effects blocked by the Cxcr4 antagonist, AMD3100^27^. There is no evidence to date whether this Cxcl12/Cxcr4 chemokine system exhibits a natural chemokine gradient in the brain and if this gradient is involved in the ethanol-induced increase in Hcrt neuronal development. Also, while there are studies directly relating another chemokine system, Ccl2/Ccr2, to the consumption of alcohol^6,28^, the possibility that the Cxcl12/Cxcr4 system mediates the ethanol-induced changes in alcohol-related behaviors has yet to be investigated.

To address these questions, the present study in zebrafish was designed to: (1) perform a more detailed anatomical analysis of the Cxcl12/Cxcr4 system, from the hypothalamus to the most anterior region of the telencephalon, as it relates normally to the development of Hcrt neurons located within and around the AH; (2) investigate the effects of embryonic ethanol exposure at a low-moderate dose on this chemokine system throughout the brain and specifically within Hcrt neurons; and (3) examine the effects of embryonic administration of a Cxcr4 antagonist on the ethanol-induced changes in neuronal development and alcohol-related behaviors.

## Materials and methods

### Animals and housing

Transgenic *Hcrt* x *Cxcr4b* zebrafish (*Danio rerio*) were generated by crossing *Hcrt:EGFP* zebrafish^29^ with a *Cxcr4b:cxcr4b-Kate2-IRES-eGFP-CaaX* transgenic line that expresses Cxcr4b fused to the monomeric red fluorescent protein Kate2 from the Cxcr4b promoter and membrane-tethered GFP from IRES^17^. Through a whole-genome duplication event, zebrafish possess paralogs for Cxcr4, designated as Cxcr4a and Cxcr4b, and also for Cxcl12, designated as Cxcl12a and Cxcl12b. Although these paralogs have similar functions^30^, we focused here on Cxcl12a/Cxcr4b based on our previous work showing that a low-moderate concentration of EtOH administered from 22 to 24 h post-fertilization (hpf) increases the number of *cxcl12a* and *cxcr4b* transcripts in whole zebrafish embryos and the developing hypothalamus^27^ and other evidence showing that the Cxcl12a/Cxcr4b system mediates the migration of hypothalamic neurons expressing the neuropeptide gonadotropin releasing hormone (GnRH)^25^. Transgenic *Hcrt x Vglut* zebrafish were generated to perform brain registration and alignment to the Zebrafish Brain Browser^31^ by crossing *Hcrt:EGFP*^29^ and *vglut2a:DsRed*^32^ transgenic lines. Zebrafish were housed with recirculating water flow at a temperature between 28 and 29 °C and a pH between 6.9 and 7.4 as described^10^. Adult zebrafish were group-housed in 3 L tanks (Aquatic Habitat, Apopka, FL), bred, and their embryos raised within an AAALAC accredited facility as described^10,27^. All protocols were approved by the Rockefeller University Institutional Animal Care and Use Committee and followed the NIH Guide for the Care and Use of Laboratory Animals. The study was carried out in compliance with the ARRIVE guidelines.

### Embryonic ethanol and AMD3100 treatment

Embryonic exposure of zebrafish to ethanol was performed as indicated in our previous reports^10,27^ and briefly summarized here. At 22 h post-fertilization (hpf), embryos were removed from an incubator and placed in a solution of either 0.0% or 0.5% (vol/vol %) ethanol, and they were then immediately returned to the incubator for 2 h, washed in fresh embryo medium, and returned to the incubator. For the Cxcr4 antagonist experiments, 1 μM of AMD3100 was prepared using embryo medium as described^33^, and the zebrafish were immersed in this solution from 2 to 24 hpf, followed by a wash in embryo medium. This low dose of AMD3100 was used since we found it to have no widespread and detrimental effects on neuronal development like that described at higher doses^34^. The 4 groups tested in this study were as follows: (1) “control”, defined as being immersed only in embryo medium at all ages; (2) “ethanol”, defined as being immersed in 0.5% v/v ethanol within embryo medium from 22 to 24 hpf; (3) “control + AMD3100”, defined as being immersed in 1 µM of AMD3100 in embryo medium from 2 to 24 hpf and; (4) “ethanol + AMD3100”, defined as being immersed in 1 µM of AMD3100 in embryo medium from 2 to 22 hpf and then immersed in 0.5% v/v ethanol and 1 μM of AMD3100 from 22 to 24 hpf.

### Zebrafish brain registration

Transgenic *Hcrt:EGFP* zebrafish at 6 days post-fertilization (dpf) were crossed with *vglut2a:DsRed* zebrafish that exhibit pan-neuronal expression and permit alignment and registration of brain images to identify the anatomical location of each Hcrt neuron, as we have previously reported^10^. We used Advanced Normalization Tools (ANTs) software^35^ on Rockefeller University’s high-performance computing cluster to perform registrations and align brains using parameters as described^31^. The registered brains of 6 dpf *Hcrt* x *Vglut* zebrafish within control, control + AMD3100, ethanol, and ethanol + AMD3100 groups were averaged and then aligned to an annotated brain atlas, the Zebrafish Brain Browser^31^, to evaluate the anatomical location of each Hcrt neuron.

### Behavioral testing

*Hcrt:EGFP* zebrafish at 8 dpf underwent behavioral testing within a DanioVision (Noldus, Wageningen, Netherlands) chamber to measure both locomotor activity and anxiety-like behaviors analyzed for the following groups: control, control + AMD3100, ethanol, and ethanol + AMD3100. Zebrafish were individually transferred from their home tanks into a standard 12-well culture plate containing fresh embryo media, and immediately placed into the behavior chamber. Zebrafish first underwent a 10-min free swimming test to assess locomotor activity by measuring distance traveled, and also thigmotaxis by measuring percentage of time spent in the perimeter of the arena to assess anxiety-like behavior^36^. Zebrafish were then returned to the incubator for 1 h of recovery, put into a new 12-well plate for a 10-min light–dark preference test to assess light preference as measured by the percentage of time spent in the light half which indicates anxiety in larval zebrafish^37^. Zebrafish activity was tracked by Noldus Ethovision XT 16 software, followed by manual correction of swim tracks if necessary.

### RNAscope in situ hybridization

We used the previously described RNAscope in situ hybridization procedure with minor changes^27,38^. Briefly, 20, 24 and 28 hpf *Hcrt*:*EGFP* zebrafish embryos were dechorionated and fixed overnight in 4% PFA with PBST (1% Tween-20) at 4 °C. The samples were then washed 3 × 5 min with 0.1% PBST, dehydrated in 25–50–75–100% methanol-0.1% PBST solution, and stored in 100% methanol at − 20 °C for at least one night. The embryos were then air-dried for 30 min at RT and subjected to the RNAscope-based signal amplification (Advanced Cell Diagnostics, Newark, CA). Protease digestion of embryos using Protease III was performed for 20 min at RT followed by rinsing the embryos three times in 0.01% PBST (0.01% Tween-20 in PBS). Hybridization of *cxcl12a* and *cxcr4b* probes was performed at 40 °C and labeled with Atto 550 and Atto 647, respectively. A probe diluent was used in one channel to permit visualization of the endogenous *Hcrt:EGFP* signal, and Amp4 Alt A was used to label the probes. Pre-amplifier hybridization (30 min), signal enhancement (15 min), amplifier hybridization (30 min), and labeling (15 min) were all conducted at 40 °C and separated by 3 × 15 min washes in 0.2 × SSCT at RT. The samples were incubated overnight at 4 °C in DAPI (1:200) diluted in 0.2% PBST. Lastly, the samples were washed 3 × 5 min with PBS and stored in fresh PBS at 4 °C until imaging.

### Immunofluorescence histochemistry

All groups of 20, 24 and 28 hpf *Hcrt* × *Cxcr4b* zebrafish embryos were fixed overnight at 4 °C in 4% PFA in PBST (1% Tween-20) as previously described^39^. Samples were washed 4 × 15 min with 1% PBST and blocked for 2 h in PBS containing 2% normal donkey serum, 0.5% Tween-20, and 1% BSA. The samples were incubated overnight at 4 °C with shaking in chicken anti-GFP (Aves Labs; 1:500) and rabbit anti-tRFP (Evrogen; 1:500) primary antibodies diluted with the blocking solution. The samples were washed 4 × 15 min with 0.1% PBST and incubated overnight at 4 °C with shaking in Alexa fluor 488 anti-chicken (Invitrogen; 1:500) and Alexa Fluor 647 anti-rabbit (Abcam; 1:500) secondary antibodies and DAPI (1:200) diluted in 0.2% PBST. Lastly, the samples were washed 4 × 15 min with 0.2% PBST and stored at 4 °C.

### Microscopy and image analysis

Transgenic 6 dpf *Hcrt* × *Vglut* zebrafish were live-imaged using confocal microscopy with a 25 × objective lens on a LSM 780, with a z step of 1.0 μm to identify the anatomical location of each Hcrt neuron and to measure their number. We analyzed 6 dpf zebrafish brain images using Imaris 9.9.1 software. The AH and preoptic area (POA) brain areas were manually cropped out of the whole brain images using the “Surface” function in Imaris following previously published guidelines^31^. After noticing differences in the location of Hcrt neurons between the anterior and posterior parts of AH in our initial analyses, we decided to quantify this difference by dividing the volume of the AH into equal halves along the AP axes into regions we termed anterior AH (aAH) and posterior AH (pAH). Hcrt neuron number in aAH and pAH as well as POA was then quantified by counting the number of each Hcrt neuron in each delineated region using the “Spots” function in Imaris.

**Figure 1:**
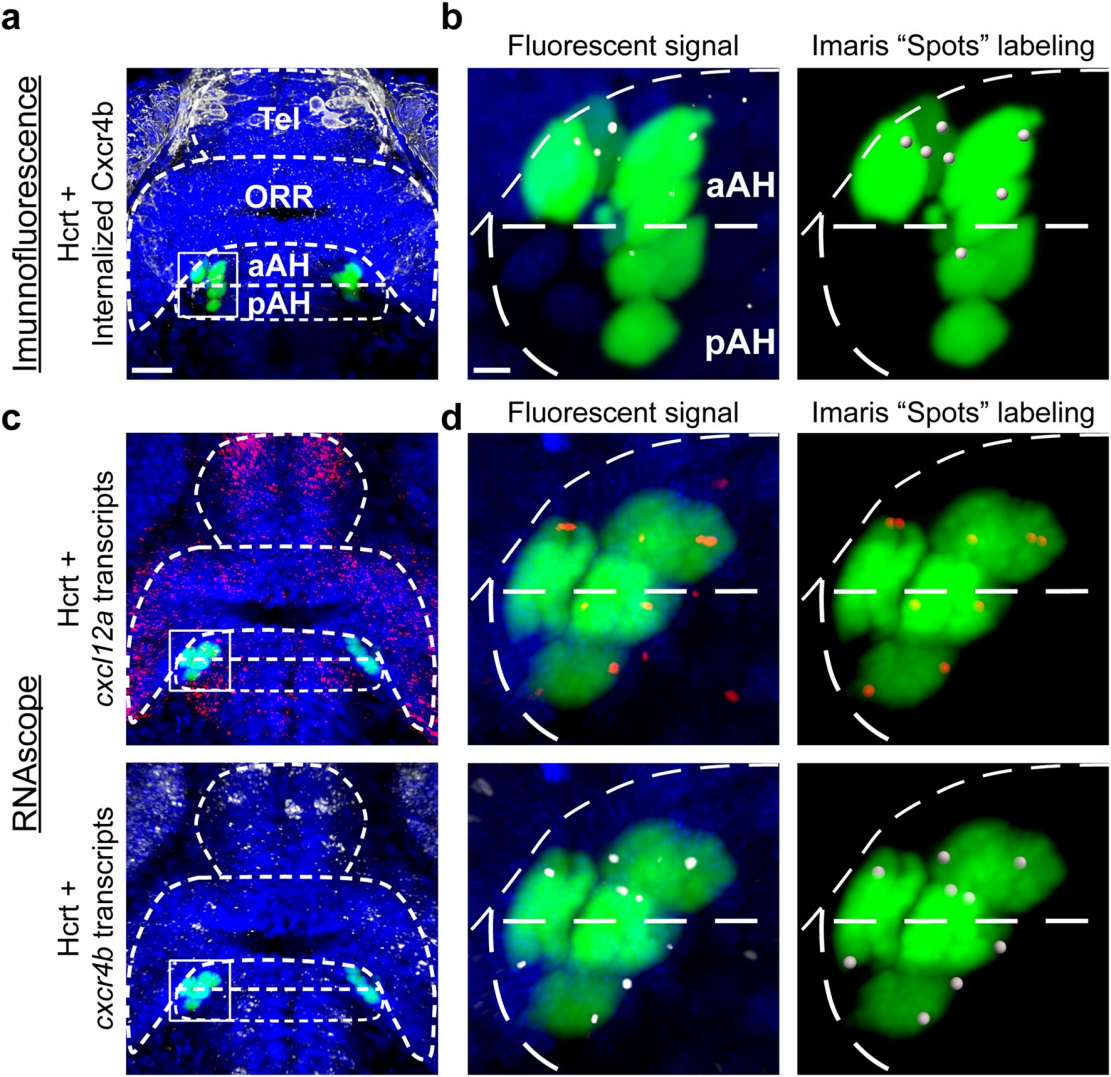
Photomicrographs illustrating the methodology used to quantify internalized Cxcr4b receptors colocalized within Hcrt neurons using immunofluorescence and also *cxcl12a* and *cxcr4b* transcripts colocalized within Hcrt neurons using RNAscope. (**A**) A representative confocal photomicrograph (×25) illustrates the 28 hpf zebrafish brain in a dorsal/ventral view with the aAH, pAH, ORR and Tel outlined by dashed lines. The Hcrt neurons (green) and internalized Cxcr4b receptors (white) are labeled using immunofluorescence, with counterstaining using DAPI (blue). (**B**) Enlargement of boxes at 28 hpf show the fluorescent signal (left) of the Hcrt neurons and internalized Cxcr4b receptors and the “Spots” labeling (right) of these internalized Cxcr4b receptors within Hcrt neurons using Imaris software for quantification. (**C**) Representative confocal photomicrographs (×25) show Hcrt neurons (green) with *cxcl12a* (top, red) and *cxcr4b* (bottom, white) transcripts labeled using RNAscope, with counterstaining using DAPI (blue). (**D**) Enlargement of boxes at 28 hpf show the fluorescent signal (left) of the Hcrt neurons and colocalized *cxcl12a* (top) and *cxcr4b* (bottom) transcripts and the Imaris “Spots” labeling (right) of these transcripts within Hcrt neurons using Imaris software for quantification. Scale bar low magnification 20 µm; high magnification 8 µm. *aAH* anterior part of the anterior hypothalamus, *pAH* posterior part of the anterior hypothalamus, *ORR* optic recess region, *Tel* telencephalon, *Hcrt* hypocretin, *hpf* hours post fertilization.

The RNAscope and Immunofluorescence (IF) samples of *Hcrt:EGFP* and *Hcrt x Cxcr4b* zebrafish at 20, 24, and 28 hpf were imaged with a 25×/1.3 objective lens on an inverted Zeiss LSM 780 laser scanning confocal microscope. 28 hpf images were then analyzed in Imaris 9.9.1 software. The AH, the optic recess region (ORR) that develops into the POA^40^, and the telencephalon (Tel) were cropped out of the whole brain images using the “Surface” function in Imaris. For RNAscope images, the number of Hcrt neurons was quantified manually and the density of *cxcl12a* and *cxcr4b* transcripts were quantified using the “Spots” function with a 1 µm spot size determined by measuring the diameter of these transcripts and then normalized to the volume exported from “Surface” of each brain region in Imaris. To measure *cxcl12a* and *cxcr4b* transcripts colocalizing with Hcrt neurons, we first applied Imaris “surface” to mask Hcrt neurons and used the “Spot colocalize with surface” function in Imaris (spot distance threshold of 1 µm) to ensure these transcripts were in fact colocalized within the masked Hcrt neurons. For IF images, we quantified the number of internalized Cxcr4b and their colocalization with Hcrt neurons by counting the number of puncta in the Cxcr4b channel using the same methodology in Imaris as described above, with this quantification being a previously reported measure of Cxcr4b receptor internalization^27^. Although these chemokine transcripts and internalized receptors are small in size and exhibit dim expression when colocalizing with neurons, Imaris software is sensitive enough to detect their expression and colocalization within these Hcrt neurons, with photomicrographs illustrating both the fluorescent signal of the internalized Cxcr4b receptors (Fig. 1a,b) and the transcripts of *cxcl12a* and *cxcr4b* (Fig. 1c,d) along with their labeling using Imaris “Spots”.

### Statistical analyses

Zebrafish live-imaging data were analyzed using two-way ANOVA, which tested the main effects of drug condition and brain area and their interactions, followed by post-hoc Tukey’s multiple comparisons test. Behavioral data were analyzed using one-way ANOVA, followed by Sidak’s post-hoc test. Data from the control only experiments using RNAscope and IF, which measured at 28 hpf the number of Hcrt, number of *cxcl12a* and *cxcr4b* transcripts in Hcrt and number of internalized Cxcr4b in Hcrt, were analyzed by unpaired *t* tests, while the density of *cxcl12a* and *cxcr4b* transcripts and internalized Cxcr4b were analyzed using one-way ANOVA, followed by post-hoc Tukey’s test. All data from RNAscope and IF experiments for control, control + AMD3100, ethanol, and ethanol + AMD3100 groups at 28 hpf were analyzed using a two-way ANOVA, which tested the main effects of drug condition and brain area and their interactions, followed by post-hoc Tukey’s multiple comparisons test. With zero Hcrt neurons evident within the POA or ORR in the control, the control + AMD3100, and the ethanol + AMD3100 groups, no statistical comparisons were made between these groups and the ethanol group for Hcrt number and colocalization data. All tests were two-tailed, and significance was determined at p < 0.05. All data were analyzed using Prism (version 8, GraphPad, San Diego, CA) and are presented as mean ± SEM in the figures and table.

## Results

### Cxcr4 receptors mediate the ethanol-induced increase in Hcrt neurons in the aAH and ectopically in the POA

**Figure 2:**
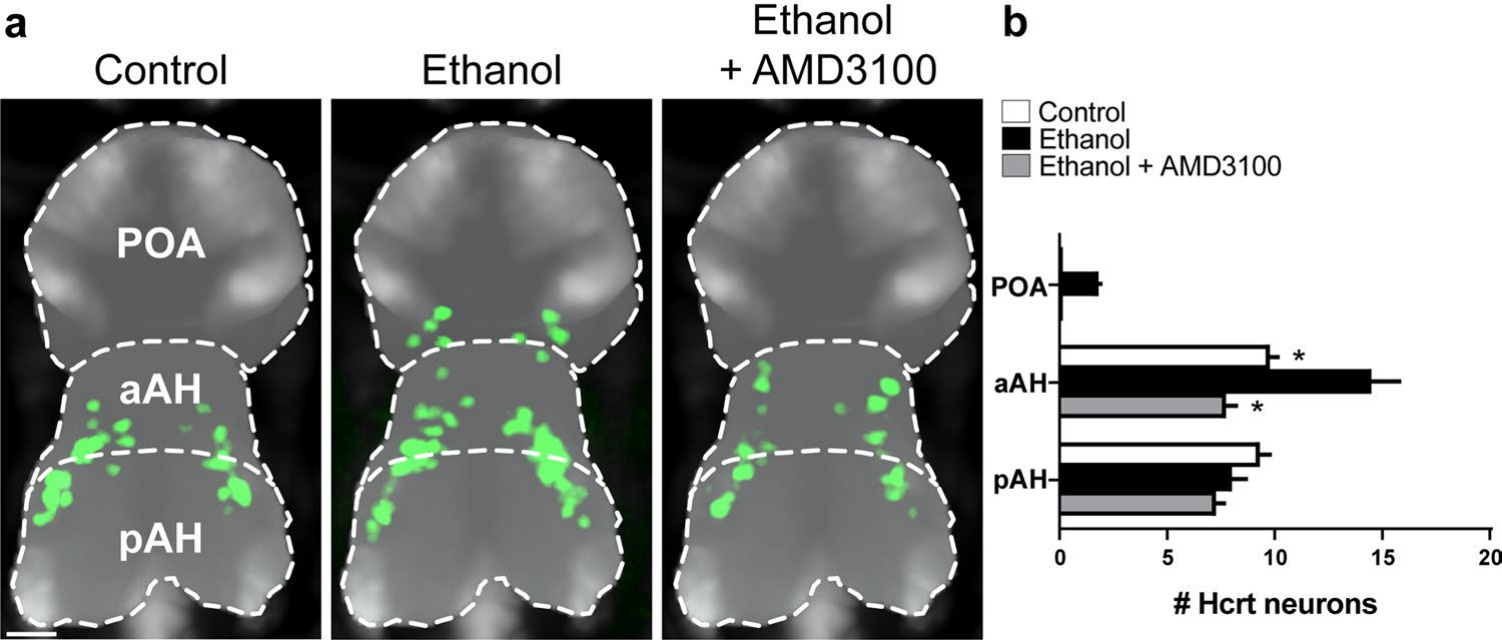
Effects of exposure to embryonic ethanol (0.5% v/v, 22–24 hpf) and Cxcr4 antagonist AMD3100 (1 µM, 2–24 hpf) on the location of Hcrt neurons in the aAH, pAH and POA of 6 dpf zebrafish brains. (**a**) Photomicrographs (×25, overlay of n = 4 images in each condition) illustrate zebrafish brains, in a dorsal/ventral view obtained at 6 dpf using live-imaging, with pan-neuronal vglut2a expression (grey) and also Hcrt neurons (green) normally located in the aAH and pAH of control, ethanol, and ethanol + AMD3100 zebrafish and ectopically located in the POA of only ethanol zebrafish. (**b**) Bar graphs (n = 4/group) show that ethanol compared to control increases the number of Hcrt neurons in the aAH and induces ectopic neurons in the POA while having no effect in the pAH, and these effects on Hcrt neurons in the aAH and POA are totally blocked by AMD3100. Scale bar 20 µm. *p < 0.05 compared to ethanol. Results are shown as means ± standard errors. *aAH* anterior part of the anterior hypothalamus, *pAH* posterior part of the anterior hypothalamus, *POA* preoptic area, *Hcrt* hypocretin, *hpf* hours post fertilization, *dpf* days post fertilization.

We first tested in zebrafish at 6 dpf if a low-moderate concentration of ethanol (0.5% v/v) alters the number and anatomical location of Hcrt neurons and if these effects of ethanol are prevented by pretreatment with the Cxcr4 antagonist, AMD3100 (1 µm). Analysis of whole-brain images of *Hcrt:EGFP* zebrafish at 6 dpf (Fig. 2a,b) revealed a main effect of both drug (*F* (3, 36) = 11.75, *p* < 0.0001) and brain area (*F* (2, 36) = 279.6, *p* < 0.0001) and an interaction between drug and brain area (*F* (6, 36) = 6.84, *p* < 0.0001). In control zebrafish, Hcrt neurons were evenly distributed throughout their normal location within the pAH and aAH and never detected anterior to the AH in the POA (Fig. 2b). Compared to control, embryonic ethanol exposure increased the number of Hcrt neurons within the AH, producing this effect specifically in the aAH (*p* < 0.0001) but not the pAH (*p* = 0.9993), and it also induced ectopic Hcrt neurons further anterior in the POA (Fig. 2b). These stimulatory effects of ethanol were totally blocked by embryonic administration of the Cxcr4 antagonist AMD3100, which prevented the ethanol-induced increase in Hcrt neurons in the aAH (*p* = 0.0003) and induction of ectopic Hcrt neurons in the POA while having no effect on Hcrt number in the pAH (*p* = 0.9568, ns) (Fig. 2b). These results demonstrate that the Cxcl12/Cxcr4 chemokine system is involved in mediating the ethanol-induced increase in Hcrt neurons in the anterior area of the AH and further anterior ectopically in the POA.

### Cxcr4 receptors mediate an ethanol-induced increase in locomotor and anxiety-like behaviors

**Figure 3:**
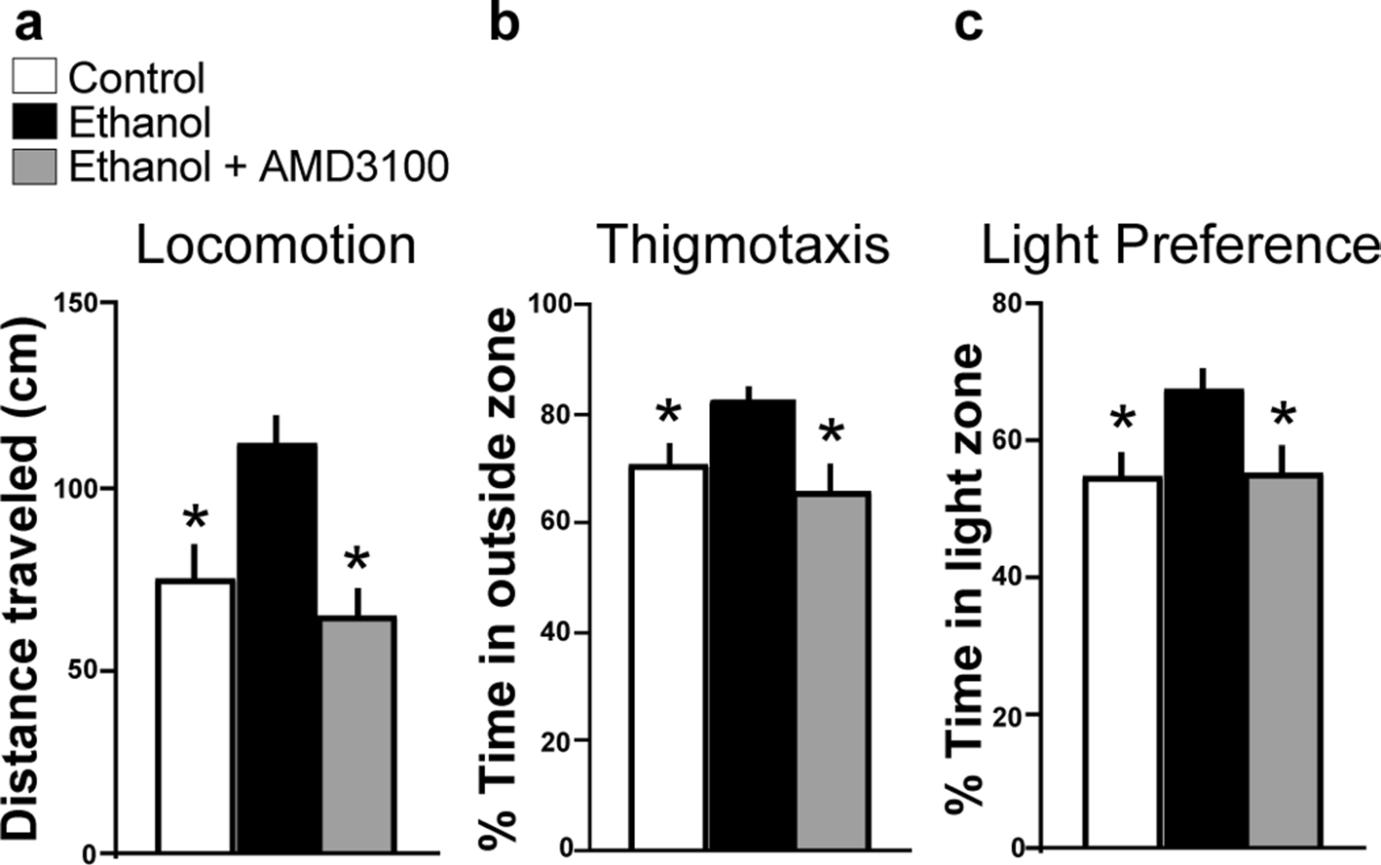
Effects of embryonic ethanol (0.5% v/v, 22–24 hpf) and Cxcr4 antagonist AMD3100 (1 µM, 2–24 hpf) on locomotor and anxiety-like behaviors in 8 dpf zebrafish. (**a**) Bar graphs (n = 29/group) show that ethanol compared to control increases locomotor activity, as indicated by an increase in distance traveled, and this ethanol effect is blocked by pretreatment with AMD3100. (**b**) Bar graphs (n = 29/group) show that ethanol increases anxiety-like behavior, indicated by an increase in perecentage of time spent in the perimeter of the arena (thigmotaxis), and this behavioral effect is blocked by AMD3100. (**c**) Bar graphs (n = 25/group) show that ethanol increases another anxiety-like behavior, indicated by an increase in percentage of time spent in the light half of the arena during the light–dark test (light preference), and this behavioral effect is blocked by AMD3100. *p < 0.05. Results are shown as means ± standard errors. *hpf* hours post fertilization, *dpf* days post fertilization.

We next tested in zebrafish at 8 dpf if the Cxcl12/Cxcr4 system is similarly involved in the behavioral changes induced by embryonic ethanol exposure, measuring both locomotor activity (distance traveled) and the anxiety-like behaviors of thigmotaxis (% time in outside zone) and light-preference (% time in light zone). There was an effect of drug on locomotor activity (*F* (3, 112) = 7.502, *p* = 0.0001), with post-hoc analysis revealing an ethanol-induced increase in locomotor activity (*p* = 0.0023) as previously reported^41^, and pretreatment with AMD3100 completely blocked this increase in locomotion (*p* < 0.0001) (Fig. 3a). In the analysis of thigmotaxis, there was also an effect of drug (*F* (3, 112) = 4.452, *p* = 0.0054), with an ethanol-induced increase in thigmotaxis (*p* = 0.0388), and a total blockade of this increase by AMD3100 (*p* = 0.0025) (Fig. 3b). Further, light-preference behavior was similarly affected, with an effect of drug (*F* (3, 96) = 3.262, *p* = 0.0248) and ethanol-induced increase in light-preference (*p* = 0.0378), and this effect was blocked by AMD3100 (*p* = 0.0450) (Fig. 3c). These behavioral results demonstrate that the Cxcl12/Cxcr4 chemokine system has a role in mediating these ethanol-induced changes in locomotor and anxiety-like behaviors.

### Posterior-to-anterior Cxcl12a/Cxcr4b concentration gradients are related to embryonic development of Hcrt neurons

**Figure 4:**
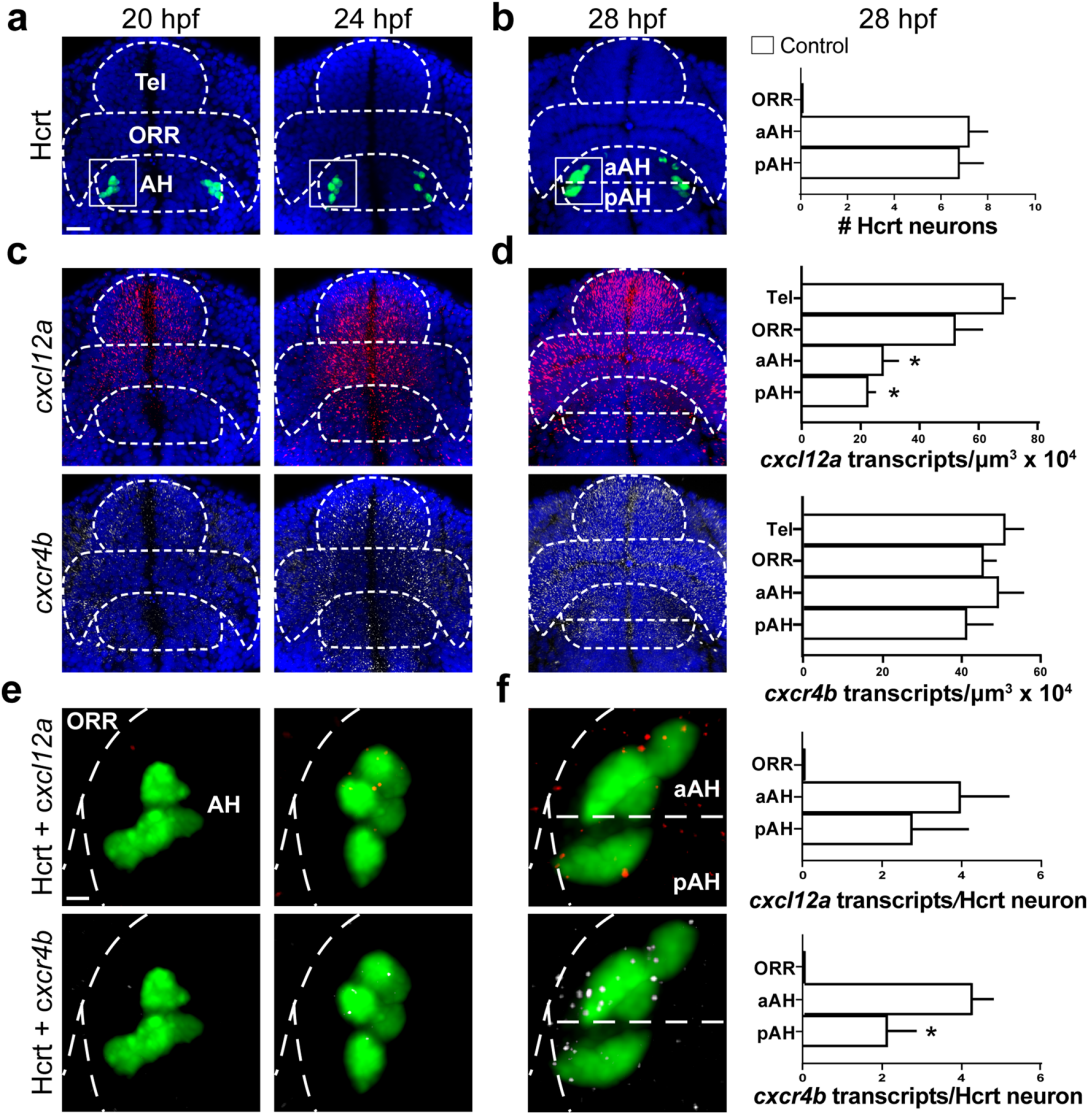
Embryonic development of Hcrt neurons and *cxcl12a* and *cxcr4b* transcripts under control conditions in 20, 24 and 28 hpf zebrafish brains, as evidenced by RNAscope. (**a**) Representative photomicrographs (×25) illustrate the zebrafish brain in a dorsal/ventral view with the AH, aAH, pAH, ORR and Tel of the brain outlined by dashed lines, the Hcrt neurons (green) tightly clustered together at 20 hpf while starting to spread out and increase in number at 24 hpf, and the cells counterstained with DAPI (blue). (**b**) Representative photomicrographs show Hcrt neurons (green) at 28 hpf that have migrated further anterior and become evenly distributed throughout the posterior (pAH) and anterior (aAH) parts of the AH, as shown in the bar graphs (n = 6). (**c**) Representative photomicrographs show the density of *cxcl12a* transcripts (red, top) and *cxcr4b* transcripts (white, bottom) naturally increases from 20 to 24 hpf, and the *cxcl12a* transcripts show a tendency to be highest in the anterior part of the brain, while the *cxcr4b* transcripts appear evenly distributed throughout all brain areas. (**d**) Representative photomicrographs show the *cxcl12a* transcripts (top) at 28 hpf as they become more dense and exhibit a natural posterior-to-anterior concentration gradient throughout the brain, with lowest levels in the pAH, moderate levels in the aAH and ORR, and highest levels in the Tel, as shown in the bar graph (n = 6). The density of *cxcr4b* transcripts (bottom), in contrast, is uniform across all brain regions, as shown in the bar graph (n = 6). (**e**) Enlargements of boxes at 20 hpf show no co-localization with *cxcl12a* (top) or *cxcr4b* (bottom) transcripts, while some co-localization is evident at 24 hpf. (**f**) Enlargement of boxes at 28 hpf show the *cxcl12a* and *cxcr4b* transcripts to exhibit high levels of co-localization with Hcrt neurons, with these colocalized transcripts showing dim expression. While the number of *cxcl12a* transcripts (top) are equally expressed in Hcrt neurons of the aAH and pAH, there is a significantly greater number of *cxcr4b* transcripts (bottom) in Hcrt neurons in the aAH compared to the pAH as shown in the bar graph (n = 6). Scale bar low magnification 20 µm; high magnification 8 µm. *p < 0.05 compared to Tel. Results are shown as means ± standard errors. *AH* anterior hypothalamus, *aAH* anterior part of the anterior hypothalamus, *pAH* posterior part of the anterior hypothalamus, *ORR* optic recess region, *Tel* telencephalon, *Hcrt* hypocretin, *hpf* hours post fertilization.

**Figure 5:**
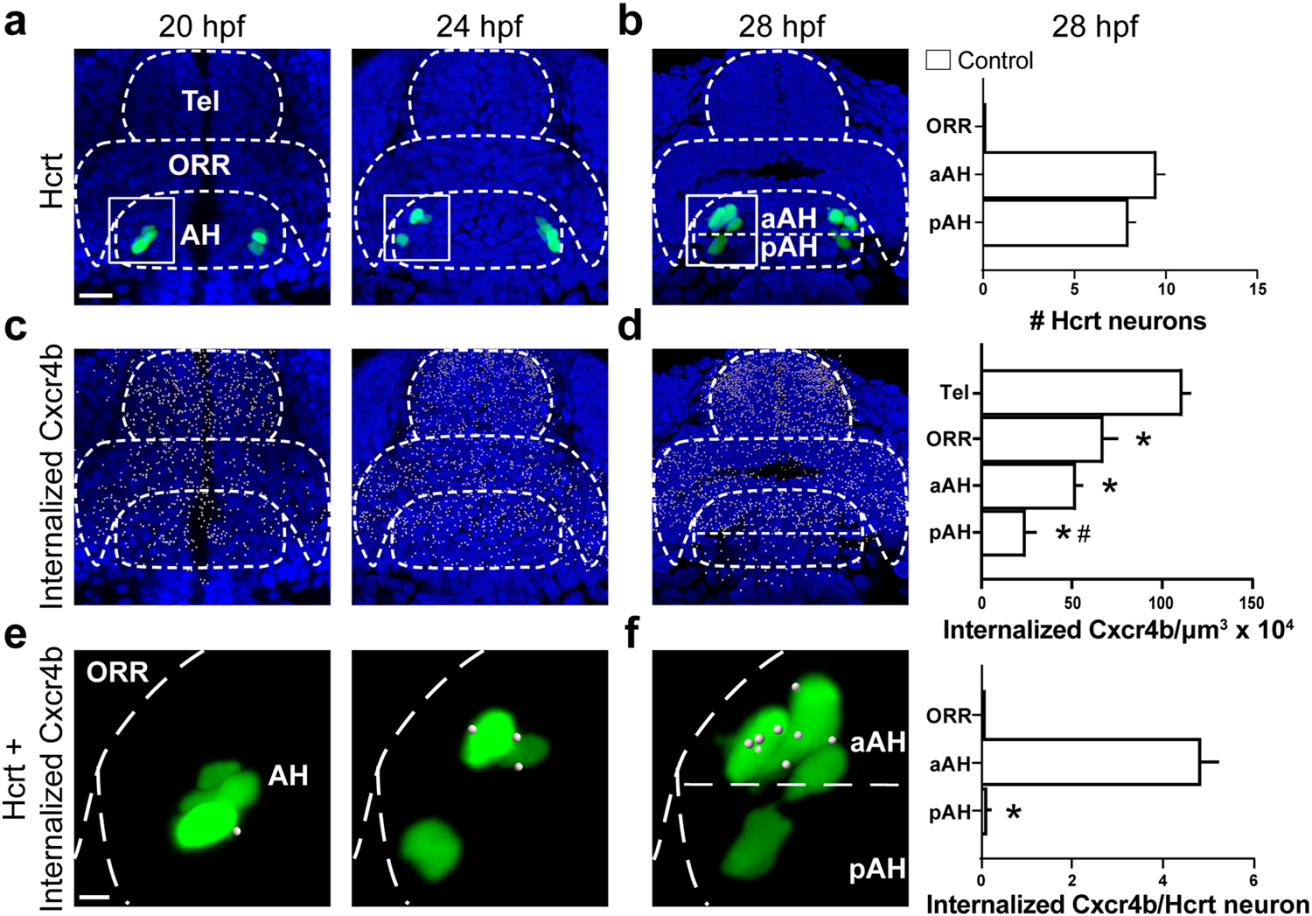
Embryonic development of Hcrt neurons and internalized Cxcr4b under control conditions in 20, 24 and 28 hpf zebrafish brains, as evidenced by immunofluorescence histochemistry. (**a**) Representative photomicrographs (×25) illustrate the zebrafish brain in a dorsal/ventral view with the pAH, aAH, ORR and Tel of the brain outlined by dashed lines, the Hcrt neurons (green) shown tightly clustered together at 20 hpf while spreading out and increasing in number at 24 hpf, and the cells counterstained with DAPI (blue). (**b**) Hcrt neurons become more spread apart at 28 hpf, with an equal number of Hcrt neurons detected in the aAH and the pAH and no Hcrt neurons detected in the ORR, as shown in the bar graph (n = 4). (**c**) Representative photomicrographs show the density of internalized Cxcr4b receptors (white, indicated by digital “spots”) naturally increases from 20 to 24 hpf, and they show a tendency to increase from the AH to the Tel part of the brain. (**d**) Representative photomicrographs show the internalized Cxcr4b receptors at 28 hpf become more dense and exhibit a natural posterior-to-anterior concentration gradient throughout the brain, with lowest levels in the pAH, moderate levels in the aAH and ORR, and highest levels in the Tel, as shown in the bar graphs (n = 4). (**e**) Enlargements of boxes at 20 hpf show no Hcrt neurons with internalized Cxcr4b receptors, while internalized Cxcr4b receptors within Hcrt neurons become apparent at 24 hpf. (**f**) Enlargement of boxes at 28 hpf show an increase in the number of internalized Cxcr4b receptors in Hcrt neurons, with Hcrt neurons located in the aAH showing a greater number than the pAH of these internalized Cxcr4b receptors, as shown in the bar graphs (n = 4). Scale bar: low magnification 20 µm; high magnification 8 µm. *p < 0.05 and ^#^*p* < 0.05 compared to Tel and aAH respectively. Results are shown as means ± standard errors. *AH* anterior hypothalamus, *aAH* anterior part of the anterior hypothalamus, *pAH* posterior part of the anterior hypothalamus, *ORR* optic recess region, *Tel* telencephalon, *Hcrt* hypocretin, *hpf* hours post fertilization.

To further understand the function of the Cxcl12a/Cxcr4b chemokine system in the differential effects of ethanol on the number and location of Hcrt neurons, we used RNAscope and IF first to examine under control conditions this chemokine system along with Hcrt as they develop normally in the embryo from 20 to 28 hpf. We measured the density of both *cxcl12a* and *cxcr4b* transcripts and internalized Cxcr4b receptors, from the pAH in the most posterior area through the aAH and ORR and into the Tel of the anterior brain, and also examined their colocalization within Hcrt neurons. Our analysis of Hcrt neurons in control zebrafish using both RNAscope (Fig. 4a,b) and IF (Fig. 5a,b) revealed at 20 hpf 6–8 Hcrt neurons in the lateral area of the developing hypothalamus and 8–10 Hcrt neurons at 24 hpf when they started to spread out and migrate to a more anterior position within the AH. This number increased to 13–15 Hcrt neurons at 28 hpf, which migrated further anterior from their site of origin and were evenly distributed throughout the pAH and aAH as shown for both RNAscope samples (t (8) = 0.3086, *p* = 0.7655, ns) (Fig. 4b) and IF samples (t (6) = 2.324, *p* = 0.0591, ns) (Fig. 5b), with no Hcrt neurons detected in the ORR.

Our analysis of *cxcl12a* and *cxcr4b* transcripts and internalized Cxcr4b receptors in control zebrafish throughout the brain showed them to increase with age from 20 to 28 hpf. The *cxcl12a* transcripts (Fig. 4c,d) and internalized Cxcr4b receptors (Fig. 5c,d) exhibited at each age a natural posterior-to-anterior concentration gradient across brain areas, with levels lowest in the pAH, somewhat higher in the aAH and ORR, and highest in the Tel. A detailed analysis at 28 hpf showed that the density of *cxcl12a* transcripts varied across these brain areas (*F* (3, 20) = 13.82, *p* < 0.0001), with levels greater in the Tel than the pAH (*p* < 0.0001) and aAH (*p* = 0.0004) but not ORR (*p* = 0.2146, ns) (Fig. 4d), and the density of internalized Cxcr4b receptors similarly varied across brain areas (*F* (3, 12) = 35.79, *p* < 0.0001), with levels greater in the Tel than the pAH (*p* < 0.0001), aAH (*p* < 0.0001), and ORR (*p* = 0.0012) and also greater in the aAH compared to the pAH (*p* = 0.0328) (Fig. 5d). Transcripts of *cxcr4b*, in contrast, exhibited no gradient and were uniformly distributed across brain regions (*F* (3, 20) = 0.6174, *p* = 0.6118, ns) (Fig. 4d).

Further analyses of the *cxcl12a* and *cxcr4b* transcripts and internalized Cxcr4b receptors colocalizing with Hcrt revealed a close relationship between this chemokine system and Hcrt neurons as they develop normally from 20 to 28 hpf. While their colocalization in control fish was not observed at 20 hpf, at 24 hpf when the Hcrt neurons started to spread out and migrate further anteriorly (Figs. 4a, 5a), we observed a low level of colocalization in Hcrt neurons with faintly expressed *cxcl12a* and *cxcr4b* transcripts (Figs. 4e,f) and internalized Cxcr4b receptors (Figs. 5e,f). The highest levels of colocalization were observed at 28 hpf when the Hcrt neurons had migrated further anterior and co-expressed significantly more *cxcr4b* transcripts in the aAH compared to the pAH (t (10) = 2.364, *p* = 0.0397) but not *cxcl12a* transcripts (t (10) = 0.6561, *p* = 0.5265, ns) (Fig. 4f). These Hcrt neurons also expressed significantly more internalized Cxcr4b receptors in the aAH than pAH (t (6) = 12.28, *p* < 0.0001) (Fig. 5f). Collectively, these results provide evidence for natural chemokine concentration gradients across the embryonic brain that are related to specific patterns of colocalization with Hcrt neurons and natural differences between the anterior and posterior areas of the AH.

### Ethanol increases *cxcl12a *and *cxcr4b* transcripts in all areas and in Hcrt neurons only in the aAH and ORR

**Figure 6:**
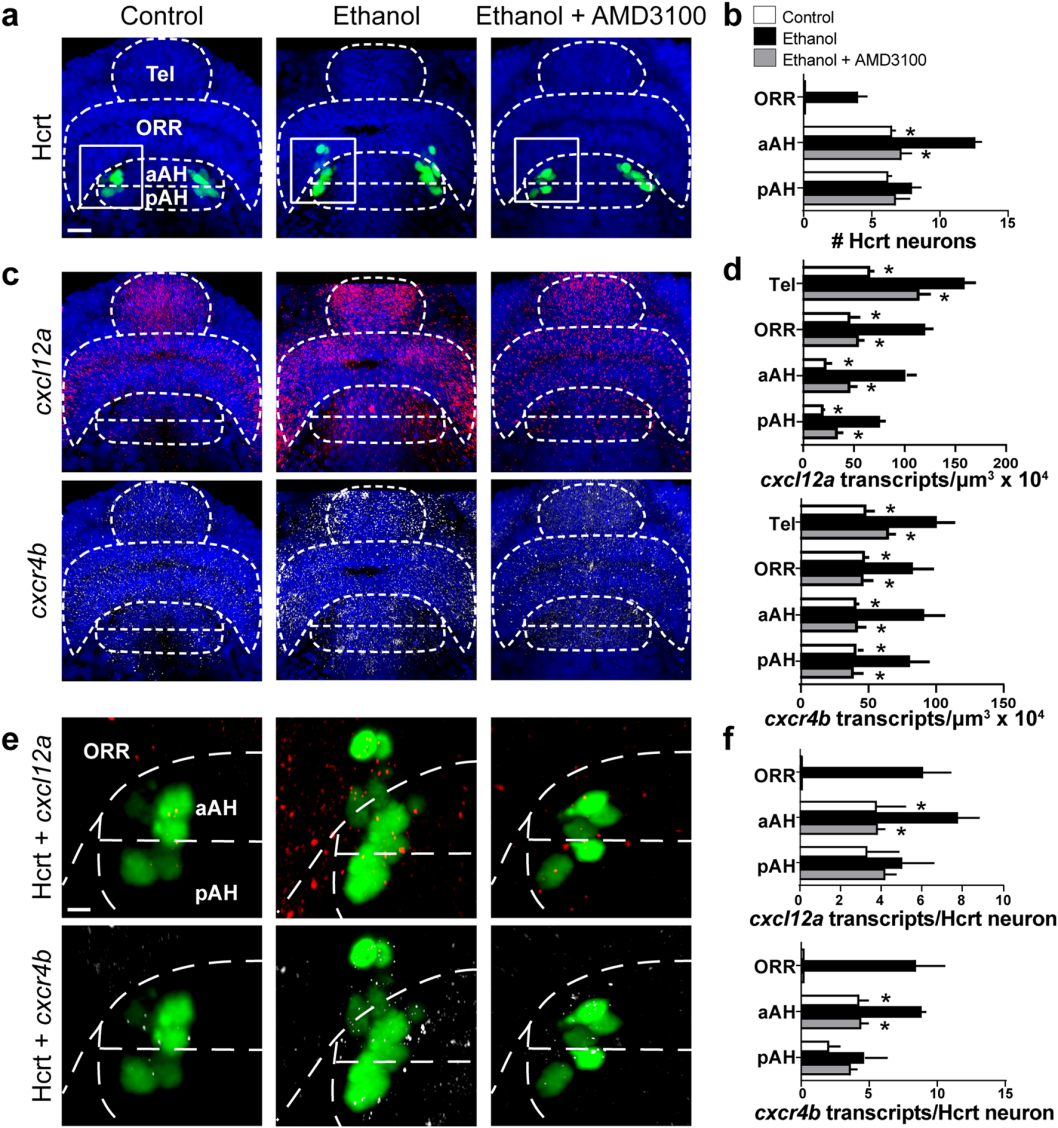
Effects of exposure to embryonic ethanol (0.5% v/v, 22–24 hpf) and administration of Cxcr4 antagonist AMD3100 (1 µM, 2–24 hpf) on the location of Hcrt neurons, density of *cxcl12a* and *cxcr4b* transcripts, and colocalization of *cxcl12a* and *cxcr4b* transcripts with Hcrt neurons in 28 hpf zebrafish brain. (**a**) Representative photomicrographs (×25) illustrate the zebrafish brain in a dorsal/ventral view with the pAH, aAH, ORR and Tel of the brain outlined by dashed lines and show that ethanol compared to control increases the number of Hcrt neurons (green) at 28 hpf only in the aAH and ectopically in the ORR, and these effects are completely blocked by AMD3100 as shown with cells counterstained with DAPI (blue). (**b**) Bar graphs (n = 4–5) show increased number of Hcrt neurons in the aAH of the ethanol group relative to the control and ethanol + AMD3100 groups, with no effect of ethanol on Hcrt number in the pAH. Ethanol-induced Hcrt neurons in the ORR are evident only in the ethanol group (**c**) Representative photomicrographs show the density of internalized *cxcl12a* (red, top) and *cxcr4b* (white, bottom) transcripts to be increased throughout the brain in the ethanol group compared to the control and ethanol + AMD3100 groups. (**d**) Bar graphs (n = 4–5) show increased density of the *cxcl12a* (top) and *cxcr4b* (bottom) transcripts in all brain regions, including the pAH, aAH, ORR and Tel, in the ethanol group compared to the control and ethanol + AMD3100 groups. (**e**) Enlargement of boxes at 28 hpf show the Hcrt neurons, located in the aAH and ectopically in the ORR but not the pAH, co-localize with a greater number of both *cxcl12a* (top) and *cxcr4b* (bottom) transcripts in the ethanol group compared to the control and ethanol + AMD3100 groups. (**f**) Bar graphs (n = 4–5) show an increased number of *cxcl12a* (top) and *cxcr4b* (bottom) transcripts in Hcrt neurons in the aAH of the ethanol group compared to the control and ethanol + AMD3100 groups, with no change between groups occurring in the pAH. Ectopically located Hcrt neurons in the ORR seen only in the ethanol group show similar levels of co-localization as evident in Hcrt neurons of the aAH. Scale bar low magnification 20 µm; high magnification 8 µm. *p < 0.05 compared to ethanol. Results are shown as means ± standard errors. *AH* anterior hypothalamus, *aAH* anterior part of the anterior hypothalamus, *pAH* posterior part of the anterior hypothalamus, *ORR* optic recess region, *Tel* Telencephalon, *Hcrt* Hypocretin, *hpf* hours post fertilization.

This experiment examined at 28 hpf using RNAscope the effects of embryonic ethanol exposure on the location of Hcrt neurons, the density of *cxcl12a* and *cxcr4b* transcripts throughout the posterior-to-anterior brain areas, and their colocalization with Hcrt neurons normally located in the pAH and aAH and ectopically in the ORR, and it also examined if the effects of ethanol are dependent on the Cxcr4 receptor and thus blocked by the antagonist, AMD3100. In our analysis of Hcrt, we observed main effects of both drug (*F* (2, 33) = 35.36, *p* = 0.0001) and brain area (*F* (2, 33) = 109.6, *p* < 0.0001) and a drug x brain area interaction (*F* (4, 33) = 4.083, *p* = 0.0085). Similar to our findings in 6 dpf zebrafish, post-hoc analysis at 28 hpf revealed an ethanol-induced increase in Hcrt number in the aAH (*p* < 0.0001) but not the pAH (*p* = 0.8896, ns) and induction of ectopic Hcrt neurons in the ORR where they are not normally detected (Fig. 6a,b). While having no effect on ethanol-exposed Hcrt neurons in the pAH (*p* = 0.7704, ns), pretreatment with AMD3100 completely blocked the ethanol-induced increase in Hcrt number in the aAH (*p* < 0.0001) and ectopic Hcrt neurons in the ORR (Fig. 6a,b).

Our analysis of *cxcl12a* and *cxcr4b* transcripts at 28 hpf showed that embryonic ethanol exposure compared to control increased *cxcl12a* transcripts throughout a posterior-to-anterior gradient, while increasing *cxcr4b* transcripts in all areas without a gradient. The analysis of *cxcl12a* transcripts revealed main effects of drug (*F* (3, 56) = 81.00, *p* < 0.0001) and brain area (*F* (3, 56) = 51.65, *p* < 0.0001) and a drug x brain area interaction (*F* (9, 56) = 2.280, *p* = 0.0295). Post-hoc analysis showed that ethanol significantly increased the density of *cxcl12a* transcripts in all areas, the pAH (*p* < 0.0001), aAH (*p* < 0.0001), ORR (*p* < 0.0001) and Tel (*p* < 0.0001) (Fig. 6c,d), and these effects of ethanol were totally blocked by AMD3100 in all areas, the pAH (*p* = 0.0013), aAH (*p* < 0.0001), ORR (*p* < 0.0001), and Tel (*p* = 0.0005) (Fig. 6c,d). Ethanol similarly increased the density of *cxcr4b* transcripts, with a main effect of drug (*F* (3, 56) = 22.91, *p* < 0.0001) but no effect of brain area (*F* (3, 56) = 0.9326, *p* = 0.4311, ns) and no drug x brain area interaction (*F* (9, 56) = 0.9314, *p* = 0.5056, ns) confirming the uniform distribution of these *cxcr4b* transcripts. Ethanol increased *cxcr4b* transcript density in all areas, the pAH (*p* = 0.0094), aAH (*p* = 0.0007), ORR (*p* = 0.024), and Tel (*p* = 0.0004) (Fig. 6c,d), and these effects were blocked in all areas by AMD3100 pretreatment, the pAH (*p* = 0.0046), aAH (*p* = 0.0010), ORR (*p* = 0.0214), and Tel (*p* = 0.0216) (Fig. 6c,d).

Further analyses of the effects of ethanol compared to control on the colocalization of *cxcl12a* and *cxcr4b* transcripts with Hcrt at 28 hpf showed higher levels of chemokine expression in Hcrt neurons only in the aAH and ectopically in the ORR but not in the pAH. Analysis of the number of *cxcl12a* transcripts colocalizing with Hcrt neurons in the aAH and pAH revealed main effects of drug (*F* (3, 42) = 11.20, *p* < 0.0001) and brain area (*F* (2, 42) = 10.57, *p* = 0.0002), with no drug x brain area interaction (*F* (6, 42) = 1.423, *p* = 0.2286, ns). These *cxcl12a* transcripts in Hcrt neurons showed an ethanol-induced increase in the aAH (*p* = 0.0327) but not the pAH (*p* = 0.6147), and the ectopic Hcrt neurons in the ORR induced by ethanol exhibited similar levels of colocalization with *cxcl12a* transcripts as the aAH neurons in the ethanol condition (Fig. 6e,f). This ethanol-induced increase in number of *cxcl12a* transcripts in Hcrt neurons was blocked by AMD3100, in the aAH (*p* = 0.0347) with no change in the pAH (*p* = 0.9335, ns), and also in the ORR where the ectopic Hcrt neurons were no longer evident (Fig. 6e,f). A similar analysis of the number of *cxcr4b* transcripts in Hcrt neurons revealed main effects of drug (*F* (3, 42) = 12.92, *p* < 0.0001) and brain area (*F* (2, 48) = 8.192, *p* = 0.001) and a drug x brain area interaction (*F* (6, 42) = 2.827, *p* = 0.0211). Ethanol increased the colocalization of *cxcr4b* transcripts in Hcrt neurons, in the aAH (*p* = 0.0302) but not pAH (*p* = 0.3593, ns), and the ectopic Hcrt neurons in the ORR had high levels of *cxcr4b* transcript colocalization similar to that seen in aAH Hcrt neurons (Fig. 6e,f). Pretreatment with AMD3100 blocked this ethanol-induced increase in *cxcr4b* transcript colocalization with Hcrt neurons, in the aAH (*p* = 0.0366) with no change in the pAH (*p* = 0.9207, ns). AMD3100 also blocked the formation of ectopic Hcrt neurons in the ORR (Figs. 6e,f). These results suggest that embryonic ethanol exposure, acting through chemokine-mediated expression and signaling, promotes the anterior migration of Hcrt neurons away from the pAH and further anterior into the aAH and ectopically into the ORR.

### Ethanol increases internalized Cxcr4b receptors in all areas and only in Hcrt neurons located in the aAH and ORR

**Figure 7:**
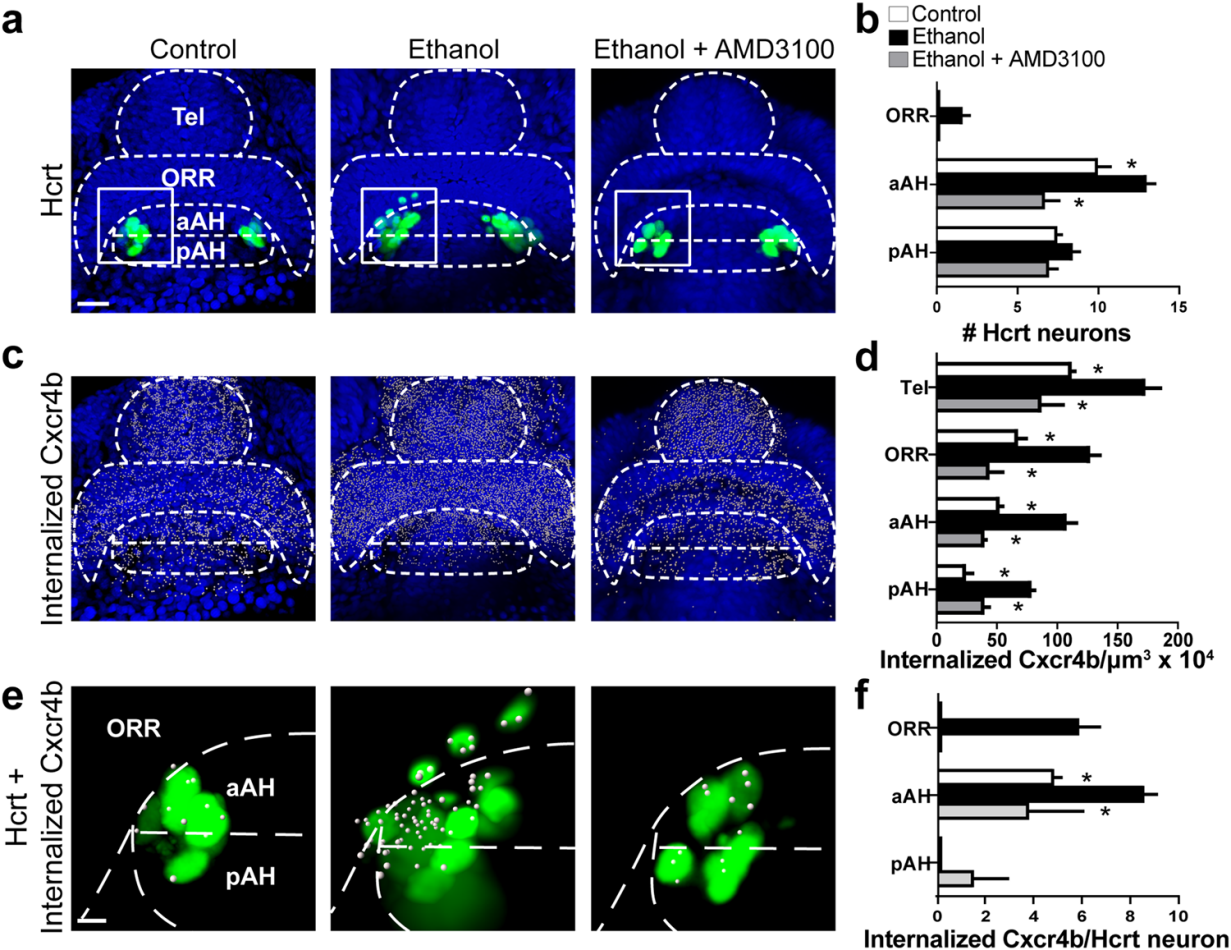
Effects of exposure to embryonic ethanol (0.5% v/v, 22–24 hpf) and administration of Cxcr4 antagonist AMD3100 (1 µM, 2–24 hpf) on the location of Hcrt neurons, the density of internalized Cxcr4b receptors, and the colocalization of internalized Cxcr4b with Hcrt neurons in the 28 hpf zebrafish brain. (**a**) Representative photomicrographs (×25) illustrate the zebrafish brain in a dorsal/ventral view with the pAH, aAH, ORR and Tel of the brain outlined by dashed lines, and show that ethanol compared to control increases the number of Hcrt neurons (green) at 28 hpf, only in the aAH and ectopically in the ORR, and these effects are completely blocked by AMD3100 as shown with cells counterstained with DAPI (blue). (**b**) Bar graphs (n = 4–5) show increased number of Hcrt neurons in the aAH of the ethanol group relative to the control and ethanol + AMD3100 groups, with no change occurring between groups in the pAH, and ectopically located Hcrt neurons located in the ORR are evident only in the ethanol group. (**c**) Representative photomicrographs show the density of internalized Cxcr4b receptors (white, indicated by digital “spots”) to be increased throughout the brain in the ethanol group compared to the control and ethanol + AMD3100 groups. (**d**) Bar graphs (n = 4–5) show increased density of internalized Cxcr4b in all brain regions including the pAH, aAH, ORR and Tel in the ethanol group relative to the control and ethanol + AMD3100 groups. (**e**) Enlargement of boxes at 28 hpf show ethanol treatment to increase the number of internalized Cxcr4b receptors in Hcrt neurons compared to both control and ethanol + AMD3100 groups. (**f**) Bar graphs (n = 4–5) show increased number of internalized Cxcr4b in Hcrt neurons in the aAH of the ethanol group compared to the control and ethanol + AMD3100 groups, with no change occurring between groups in the pAH, and ectopically located Hcrt neurons in the ORR of the ethanol group show a similar number of internalized Cxcr4b in Hcrt neurons relative to those in the aAH. Scale bar low magnification 20 µm; high magnification 8 µm. *p < 0.05 compared to ethanol. Results are shown as means ± standard errors. *AH* anterior hypothalamus, *aAH* anterior part of the anterior hypothalamus, *pAH* posterior part of the anterior hypothalamus, *ORR* optic recess region, *Tel* Telencephalon, *Hcrt* Hypocretin, *hpf* hours post fertilization.

This experiment examined at 28 hpf using IF the effects of embryonic ethanol exposure on the location of Hcrt neurons, the density of internalized Cxcr4b receptors throughout the posterior-to-anterior brain areas, and their colocalization with Hcrt neurons normally located in the pAH and aAH and ectopically in the ORR, and it also tested the effects of AMD3100 pretreatment on these ethanol-induced neuronal effects. Confirming our findings in both 6 dpf and 28 hpf zebrafish, embryonic ethanol exposure had brain-region specific effects on the number of Hcrt neurons, with main effects of drug (*F* (3, 39) = 14.96, *p* < 0.0001) and brain area (*F* (2, 39) = 246.4, *p* < 0.0001) and a drug x brain area interaction (*F* (6, 39) = 8.285, *p* < 0.0001). There was an increase in Hcrt number in the aAH (*p* = 0.0046) but not pAH (*p* = 0.7021, ns) and an induction of ectopic Hcrt neurons in the ORR (Fig. 7a,b), and pretreatment with AMD3100 completely blocked this ethanol-induced increase in Hcrt number in the aAH (*p* < 0.0001) and prevented the formation of ectopic neurons in the ORR, while producing no change in Hcrt number in the pAH (*p* = 0.3456, ns) (Fig. 7a,b).

Similar to the results for *cxcl12a* transcript density using RNAscope, we found that embryonic ethanol exposure increased Cxcr4b internalization throughout the brain without altering the natural posterior-to-anterior gradient. Analysis of the density of internalized Cxcr4b receptors revealed main effects of drug (*F* (3, 52) = 64.77, *p* < 0.0001) and brain area (*F* (3, 52) = 35.60, *p* < 0.0001) but no drug x brain area interaction (*F* (9, 52) = 1.117, *p* = 0.3697). There was an ethanol-induced increase in density of internalized Cxcr4b across all areas, in the pAH (*p* = 0.0022), aAH (*p* = 0.0014), ORR (*p* = 0.0004) and Tel (*p* = 0.0003), and AMD3100 completely blocked these effects of ethanol in all areas, the pAH (*p* = 0.0392), aAH (*p* < 0.0001), ORR (*p* < 0.0001), and Tel (*p* < 0.0001) (Fig. 7c,d).

Further, embryonic ethanol produced an increase in number of internalized Cxcr4b receptors in Hcrt neurons in an area-specific manner, with main effects of drug (*F* (2, 26) = 9.472, *p* = 0.0008) and brain area (*F* (2, 26) = 22.63, *p* < 0.0001) and a drug x brain area interaction (*F* (4, 26) = 4.281, *p* = 0.0086). There was an increase in Cxcr4b internalization within Hcrt neurons in the aAH (*p* = 0.0309) while having no effect in the pAH (*p* > 0.9999, ns). Additionally, the ectopic Hcrt neurons in the ORR exhibited a comparable level of Cxcr4b internalization to those in the aAH (Fig. 7e,f). Further, this ethanol-induced internalization of Cxcr4b receptors in Hcrt neurons was completely blocked by AMD3100, specifically in the aAH (*p* = 0.0052) and ORR (*p* = 0.0017), while having no effect on Hcrt neurons in the pAH (*p* = 0.538, ns) (Fig. 7e,f). These results suggest that the internalized Cxcr4b receptors stimulated by ethanol play an important role in guiding the anterior migration of Hcrt neurons away from the pAH and further anterior into the aAH and ectopically into the ORR.

## Discussion

This report provides new evidence in zebrafish that the stimulatory effect of embryonic ethanol exposure at a low-moderate dose on Hcrt neuron number, while occurring in the AH where these neurons are normally concentrated as previously described^10^, produces this increase in Hcrt number in an anatomically distinct manner, in the anterior (aAH) but not posterior (pAH) part of this region and further anterior in the POA where they do not normally exist. These anatomically specific effects suggest that ethanol is directing the migration of these neurons in a more anterior direction, consistent with evidence that ethanol at a low dose promotes premature cell migration in the brain of zebrafish^10^ as well as rats^9^. The possibility that these differentially responsive Hcrt neurons anatomically close within the AH have different functions is supported by evidence that motivation for another drug, cocaine, is reduced by knockdown of Hcrt neurons within the lateral hypothalamic area but unaffected by knockdown of those within the more medial perifornical area^42^. Our results with embryonic administration of the Cxcr4 antagonist AMD3100 provide the first evidence directly supporting the involvement of Cxcr4 signaling in ethanol’s anatomically specific effects on Hcrt neurons, showing that AMD3100 completely blocks ethanol’s stimulatory effect on normally located neurons in the aAH and ectopically-located neurons further anterior in the POA while having no effects in the pAH. Although Cxcr4 signaling is well known to be involved in a number of important neurodevelopmental processes such as neurogenesis^43^ and neuronal migration^19,20^, this is the first report providing direct evidence that the Cxcl12a/Cxcr4b system stimulated by ethanol exposure is actively involved in promoting the formation of ectopic neurons.

Our results are also the first to demonstrate a key role for the Cxcl12a/Cxcr4b chemokine system in mediating the behavioral changes induced by embryonic ethanol exposure. The ethanol-induced increase in both locomotor activity and the anxiety-like behaviors of thigmotaxis and light-preference in 8 dpf zebrafish was totally blocked by embryonic pretreatment with the Cxcr4 antagonist, AMD3100. These behaviors that are related to alcohol consumption^44,45^ are shown to be positively linked to Hcrt neurons in zebrafish^5,46^ as well as rats^1,47^ and to be stimulated by central administration of Hcrt itself^5,48^. There is also evidence in rodents directly relating chemokines and their receptors to these behaviors, with prenatal administration of Cxcl12 into the third ventricle shown to induce novelty-induced locomotor activity and anxiety-like behaviors^49^, deletion of the chemokine receptor Ccr2 reducing preference for ethanol^28^, and antagonism of the Cxcr4 receptor blocking cocaine-induced locomotor activity and conditioned place preference^50^. Together with these findings, our results in zebrafish demonstrate the importance of the Cxcr4 chemokine receptor in the behavioral changes induced by embryonic ethanol exposure.

Our investigation here of the Cxcl12a/Cxcr4b system as it develops naturally throughout the embryonic zebrafish brain reveals its close relationship to the Hcrt neurons as they proliferate and migrate through the hypothalamus. Under control conditions, we find that Hcrt neurons first appearing at 20 hpf and increasing in number from 24 to 28 hpf migrate anteriorly and become concentrated in the AH and evenly distributed throughout the aAH and pAH, while never appearing in the ORR from which the POA develops^40^. With migrating neurons reported to co-express chemokines^51^, our findings under normal conditions are notable in linking the timing of the colocalization of *cxcl12a* and *cxcr4b* transcripts and internalization of Cxcr4b receptors within Hcrt neurons with the early changes in number and location of developing Hcrt neurons. While the Hcrt neurons at 20 hpf are tightly clustered, have yet to migrate, and exhibit no colocalization with *cxcl12a* and *cxcr4b* transcripts or internalized Cxcr4b receptors, when these neurons increase in number and start to spread out and migrate anteriorly at 24 hpf they begin to express some of these transcripts and receptors, and this colocalization increases more at 28 hpf as they migrate even further anterior.

Our quantitative analysis at 28 hpf throughout the brain of the *cxcl12a* transcripts and internalized Cxcr4b receptors, known to reflect Cxcl12a signaling^17^, shows that, while naturally increasing with age in all areas from 20 to 28 hpf and being most highly concentrated in the Tel as described^25,52^, this chemokine and its internalized receptor both exhibit strong posterior-to-anterior concentration gradients, with lowest levels in the pAH, somewhat higher levels in the aAH and ORR, and peak levels in the Tel. Our findings under control conditions suggest that these chemokine gradients, possibly formed by higher posterior expression levels of Cxcr7 that acts as a clearance receptor for removing extracellular Cxcl12 by endocytosis^18^, contribute normally to the anterior migration of Hcrt neurons. While the expression of *cxcr4b* transcripts did not exhibit a concentration gradient likely due in part to the widespread expression of this receptor in a variety of cell types^30^, our results clearly demonstrate that Hcrt neurons developing normally in the aAH express significantly higher levels of *cxcr4b* receptor transcripts than those in the pAH, likely reflecting their stronger attraction to the high levels of *cxcl12a* transcripts and signaling in the anterior brain. This idea is substantiated by reports in rodents showing gradients of Cxcl12 to guide the migration of Cxcr4-expressing neurons within forebrain structures, including GABA-ergic interneurons migrating from deeper to superficial layers of the cortex^15^ and granule cell precursors migrating from the lateral ventricle to the dentate gyrus of the hippocampus^16^. Our findings here demonstrate, under normal conditions, that the concentration gradient of Cxcl12a/Cxcr4b throughout the brain is closely related to the natural development of a localized population of Hcrt neurons within the hypothalamus.

With zebrafish allowing us to examine the whole brain during its development, we provide here the first quantitative evidence for how embryonic ethanol exposure affects the Cxcl12a/Cxcr4 system throughout the brain and how this wide-ranging effect relates to the development of hypothalamic Hcrt neurons. We demonstrate that ethanol increases the density of *cxcl12a* transcripts and internalized Cxcr4b receptors similarly throughout the entire brain while still maintaining their natural posterior-to-anterior concentration gradients. This leads us to consider the possibility that this ethanol-induced increase in chemokine levels in more anterior brain areas contributes to the increase in Hcrt neurons normally located in the aAH as well as ectopically located further anterior in the ORR, by guiding these neurons anteriorly along the posterior-to-anterior gradient. This idea is supported by our findings that Hcrt neurons located in the anterior areas of the aAH and ORR, as compared to posterior Hcrt neurons in the pAH, express significantly greater levels of *cxcr4b* transcripts and internalized Cxcr4b receptors that likely increase their attraction to *cxcl12a* in the anterior brain. It receives further support from the evidence that transplanted Cxcl12a expression in the anterior brain of zebrafish causes increased anterior migration of Cxcr4-expressing trigeminal sensory neurons^24^ and the internalization of Cxcr4b activates downstream signaling pathways related to cell proliferation and migration^53^. Together with these findings, our results demonstrate that the ethanol-induced increase in this chemokine system plays a key role in promoting the anterior migration of Hcrt neurons and their ectopic positioning.

While a number of studies have shown that the Cxcr4 antagonist AMD3100 inhibits neuronal migration^21,54^, this is the first report showing that this receptor antagonist prevents the anatomically-specific changes in neuronal development induced by embryonic ethanol exposure. The direct involvement of the Cxcl12/Cxcr4 chemokine system in ethanol’s effects on neuronal location is supported by our finding that embryonic pretreatment with AMD3100 completely blocks the ethanol-induced increase both in *cxcl12a* and *cxcr4b* transcripts and internalization of Cxcr4b throughout the entire brain and specifically within Hcrt neurons located normally in the aAH and ectopically in the ORR. This blockade of ethanol-induced ectopic Hcrt neurons, which we find to be associated with a reduction in *cxcl12a* levels in anterior regions, likely reduces the chemoattraction of these neurons towards these areas. This may involve different molecular mechanisms including the inhibition of nuclear translocation of NFκB that reduces chemokine transcription as shown in retinal progenitor cell culture^55^ and of other downstream chemokine signaling pathways associated with transcription and migration^56^. Interestingly, in contrast to the Cxcl12 gradient in mouse bone marrow endothelium reported to be reversed by AMD3100^57^, we find here in zebrafish brain that the posterior-to-anterior gradients of *cxcl12a* transcripts and internalized Cxcr4b receptors are still present after AMD3100 treatment as well as after ethanol exposure. Together, our results reported here with the AMD3100 antagonist provide strong evidence that these natural gradients play a direct role in the ethanol-induced increase in Hcrt neuron number and their anterior and ectopic location.

In summary, this study demonstrates that the Cxcl12a/Cxcr4b chemokine system mediates the stimulatory effects of embryonic ethanol exposure at a low-moderate concentration on ectopically located Hcrt neurons and Hcrt-related behaviors early in development. It shows for the first time that this chemokine and its receptor form a natural posterior-to-anterior gradient throughout the brain associated with normal Hcrt development, ethanol exposure increases chemokine expression along this gradient and also within Hcrt neurons in anterior and ectopic brain areas, and the ethanol-induced formation of ectopic Hcrt neurons and disturbances in alcohol-related behaviors are blocked by pretreatment with a Cxcr4 antagonist. With ectopic neurons and heterotopias known to be associated with neurodevelopmental disorders^58^ including FASD^12^, these findings indicate that neuronal chemokines are strongly affected by embryonic ethanol exposure and have an important function in mediating both the neuronal and behavioral changes induced by ethanol.

## Supplementary Information

Supplementary Information.

## Data Availability

The datasets used and/or analyzed during the current study are available from the corresponding author on reasonable request.
