# Supplementary material for: Neuronal chemokine concentration gradients mediate effects of embryonic ethanol exposure on ectopic hypocretin/orexin neurons and behavior in zebrafish
Source: Sci Rep. 2023 Jan 26;13:1447. doi: 10.1038/s41598-023-28369-7 (PMC9880007; doi:10.1038/s41598-023-28369-7)
Supplement: Supplementary file 1 — Supplementary Information. [file 41598_2023_28369_MOESM1_ESM.docx]

SUPPLEMENTARY INFORMATION

**Neuronal chemokine concentration gradients mediate effects of embryonic ethanol exposure**

**on ectopic hypocretin/orexin neurons and behavior in zebrafish.**

Adam D. Collier^1^, Nushrat Yasmin^1^, Olga Karatayev^1^, Abdul R. Abdulai^1^, Boyi Yu^1^, Nailya Khalizova^1^, Milisia Fam^1^,

Sarah F. Leibowitz^1*^

^1^Laboratory of Behavioral Neurobiology, The Rockefeller University, New York, NY

*Address for Correspondence:

Sarah F. Leibowitz,

Laboratory of Behavioral Neurobiology,

The Rockefeller University,

1230 York Avenue, New York, NY 10065, USA.

Phone: 212-327-8378, Fax: 212-327-8447,

E-mail: [leibow@rockefeller.edu](mailto:leibow@rockefeller.edu)

**Supplementary Table S1.** No effects on Hcrt neurons of Cxcr4 antagonist AMD3100 (1 µM, 2-24 hpf) alone in control 6 dpf zebrafish compared to control zebrafish without antagonist.

| **Brain Area** | **Measures** | **Control** | **Control + AMD3100** | ***p* Value** |
| --- | --- | --- | --- | --- |
| **POA** | **Number of Hcrt Neurons** | 0 | 0 | NA |
| **aAH** | **Number of Hcrt Neurons** | 7.75 ± 0.479 | 10.25 ± 0.479 | 0.2256 |
| **pAH** | **Number of Hcrt Neurons** | 7.250 ± 0.479 | 10 ± 1.080 | 0.1294 |

*Data are represented as mean ± SEM.*

**Supplementary Table S2.** No effects on behaviors of Cxcr4 antagonist AMD3100 (1 µM, 2-24 hpf) alone in control 8 dpf zebrafish compared to control zebrafish without antagonist.

| **Measures** | **Control** | **Control + AMD3100** | ***p* Value** |
| --- | --- | --- | --- |
| **Locomotion**  **Distance Traveled**  (cm) | 75.39 ± 9.133 | 70.18 ± 6.503 | 0.9537 |
| **Thigmotaxis**  **(Time in Outside Zone)**  (%) | 70.93 ± 3.333 | 67.93 ± 3.260 | 0.9104 |
| **Light Preference**  **(Time in Light Zone)**  (%) | 55.14 ± 3.176 | 52.71 ± 4.880 | 0.9578 |

*Data are represented as mean ± SEM.*

**Supplementary Table S3.** No effects on Hcrt neurons and *cxcl12a* and *cxcr4b* transcripts of Cxcr4 antagonist AMD3100 (1 µM, 2-24 hpf) alone in control 28 hpf zebrafish compared to control zebrafish without antagonist.

| **Brain Area** | **Measures** | **Control** | **Control + AMD3100** | ***p* Value** |
| --- | --- | --- | --- | --- |
| **Tel** | **Number of Hcrt Neurons** | 0 | 0 | NA |
|  | **Density of *cxcl12a* transcripts**  (transcripts/μm^3^ x 10^4^) | 64.363 ± 4.633 | 64.270 ± 7.950 | 0.9999 |
|  | **Density of c*xcr4b* transcripts**  (transcripts/μm^3^ x 10^4^) | 48.795 ± 6.898 | 37.462 ± 4.127 | 0.8249 |
| **ORR** | **Number of Hcrt Neurons** | 0 | 0 | NA |
|  | **Density of *cxcl12a* transcripts**  (transcripts/μm^3^ x 10^4^) | 41.146 ± 9.621 | 50.396 ± 11.507 | 0.8393 |
|  | **Density of c*xcr4b* transcripts**  (transcripts/μm^3^ x 10^4^) | 46.399 ± 3.963 | 51.932 ± 11.200 | 0.9748 |
| **aAH** | **Number of Hcrt Neurons** | 7.2 ± 0.8 | 5.5 ± 0.5 | 0.6535 |
|  | **Density of *cxcl12a* transcripts**  (transcripts/μm^3^ x 10^4^) | 26.425 ± 5.329 | 28.262 ± 4.507 | 0.9984 |
|  | **Density of c*xcr4b* transcripts**  (transcripts/μm^3^ x 10^4^) | 42.437 ± 2.819 | 50.613 ± 4.442 | 0.9249 |
| **pAH** | **Number of Hcrt Neurons** | 6.8 ± 1.02 | 5 ± 0.408 | 0.5730 |
|  | **Density of *cxcl12a* transcripts**  (transcripts/μm^3^ x 10^4^) | 21.213 ± 2.546 | 29.512 ± 5.732 | 0.8779 |
|  | **Density of c*xcr4b* transcripts**  (transcripts/μm^3^ x 10^4^) | 42.588 ± 6.462 | 51.101 ± 8.673 | 0.9163 |

*Data are represented as mean ± SEM.*

**Supplementary Table S4.** No effects on number of *cxcl12a* and *cxcr4b* transcripts per Hcrt neuron of Cxcr4 antagonist AMD3100 (1 µM, 2-24 hpf) alone in control 28 hpf zebrafish compared to control zebrafish without antagonist.

| **Brain Area** | **Measures** | **Control** | **Control + AMD3100** | ***p* Value** |
| --- | --- | --- | --- | --- |
| **ORR** | **Number of *cxcl12a* transcripts per Hcrt Neuron** | 0 | 0 | NA |
|  | **Number of c*xcr4b* transcripts per Hcrt Neuron** | 0 | 0 | NA |
| **aAH** | **Number of *cxcl12a* transcripts per Hcrt Neuron** | 3.796 ± 1.448 | 2.708 ± 0.336 | 0.8600 |
|  | **Number of c*xcr4b* transcripts per Hcrt Neuron** | 4.108 ± 0.836 | 3.855 ± 2.376 | 0.9988 |
| **pAH** | **Number of *cxcl12a* transcripts per Hcrt Neuron** | 3.348 ± 1.565 | 3.313 ± 1.152 | 0.9999 |
|  | **Number of c*xcr4b* transcripts per Hcrt Neuron** | 2.055 ± 1.187 | 4.875 ± 1.853 | 0.3597 |

*Data are represented as mean ± SEM.*

**Supplementary Table S5.** No effects on Hcrt neurons, internalized Cxcr4b receptors and number of internalized Cxcr4b per Hcrt neuron of Cxcr4 antagonist AMD3100 (1 µM, 2-24 hpf) alone in control 28 hpf zebrafish compared to control zebrafish without antagonist.

| **Brain Area** | **Measures** | **Control** | **Control + AMD3100** | ***p* Value** |
| --- | --- | --- | --- | --- |
| **Tel** | **Number of Hcrt Neurons** | 0 | 0 | NA |
|  | **Density of internalized Cxcr4b**  (dots/μm^3^ x 10^4^) | 111.249 ± 4.850 | 78.282 ± 19.098 | 0.2099 |
|  | **Number of internalized Cxcr4b per Hcrt Neuron** | 0 | 0 | NA |
| **ORR** | **Number of Hcrt Neurons** | 0 | 0 | NA |
|  | **Density of internalized Cxcr4b**  (dots/μm^3^ x 10^4^) | 67.106 ± 8.308 | 39.650 ± 17.841 | 0.3627 |
|  | **Number of internalized Cxcr4b per Hcrt Neuron** | 0 | 0 | NA |
| **aAH** | **Number of Hcrt Neurons** | 10 ± 0.816 | 8.25± 0.25 | 0.1694 |
|  | **Density of internalized Cxcr4b**  (dots/μm^3^ x 10^4^) | 52.127 ± 4.294 | 24.219 ± 7.379 | 0.3483 |
|  | **Number of internalized Cxcr4b per Hcrt Neuron** | 14.750 ± 2.658 | 4.120 ± 2.069 | 0.9622 |
| **pAH** | **Number of Hcrt Neurons** | 7.5 ± 0.289 | 9.5 ± 0.866 | 0.0926 |
|  | **Density of internalized Cxcr4b**  (dots/μm^3^ x 10^4^) | 24.635 ± 5.961 | 21.155 ± 11.346 | 0.9968 |
|  | **Number of internalized Cxcr4b per Hcrt Neuron** | 0.500 ± 0.289 | 0 | 0.9996 |

*Data are represented as mean ± SEM.*
